# Transcript Profiling of *Elf5^+/−^* Mammary Glands during Pregnancy Identifies Novel Targets of Elf5

**DOI:** 10.1371/journal.pone.0013150

**Published:** 2010-10-07

**Authors:** Renee L. Rogers, Isabelle Van Seuningen, Jodee Gould, Paul J. Hertzog, Matthew J. Naylor, Melanie A. Pritchard

**Affiliations:** 1 Centre for Functional Genomics and Human Disease, Monash Institute of Medical Research, Clayton, Victoria, Australia; 2 Inserm, U837, Centre de Recherche Jean-Pierre Aubert, Lille, France; 3 Cancer Research Program, Garvan Institute of Medical Research, Darlinghurst, New South Wales, Australia; 4 St. Vincent's Hospital Clinical School, Faculty of Medicine, University of New South Wales, Sydney, New South Wales, Australia; Cincinnati Children's Hospital Medical Center, United States of America

## Abstract

**Background:**

Elf5, an epithelial specific Ets transcription factor, plays a crucial role in the pregnancy-associated development of the mouse mammary gland. *Elf5^−/−^* embryos do not survive, however the *Elf5^+/−^* mammary gland displays a severe pregnancy-associated developmental defect. While it is known that Elf5 is crucial for correct mammary development and lactation, the molecular mechanisms employed by Elf5 to exert its effects on the mammary gland are largely unknown.

**Principal Findings:**

Transcript profiling was used to investigate the transcriptional changes that occur as a result of Elf5 haploinsufficiency in the *Elf5* ^+/−^ mouse model. We show that the development of the mouse *Elf5* ^+/−^ mammary gland is delayed at a transcriptional and morphological level, due to the delayed increase in Elf5 protein in these glands. We also identify a number of potential Elf5 target genes, including *Mucin 4*, whose expression, is directly regulated by the binding of Elf5 to an *Ets* binding site within its promoter.

**Conclusion:**

We identify novel transcriptional targets of Elf5 and show that Muc4 is a direct target of Elf5, further elucidating the mechanisms through which Elf5 regulates proliferation and differentiation in the mammary gland.

## Introduction

The mammary gland is one of a few organs able to undergo repeated phases of growth, differentiation and regression. At the onset of puberty in the female, the increase in ovarian steroids induces elongation and side-branching of the rudimentary mammary ductal system which formed embryonically. Club-shaped terminal end buds (TEBs) develop at the ends of the developing ducts which consist of a layer of cap cells at the proceeding edge and multiple inner body cell layers. These TEBs are the proliferating edge of the ducts penetrating the mammary fat pad and regress once the development of the ductal tree is complete. Some differentiation of the ductal system occurs at this stage, resulting in a compact glandular structure. The gland then remains relatively inactive until a pregnancy occurs [reviewed in 1], [ 2].

Lobuloalveolar development commences with the onset of pregnancy and is associated with the formation of spherical alveoli along the ductal network that formed during puberty. By the end of pregnancy, the ductal tree is densely populated with alveoli. Functional differentiation of the alveoli commences during late pregnancy and this process is complete by parturition to enable lactation. The mammary gland continues to produce milk until weaning, at which time the gland regresses in a process known as involution. This cyclical development of the mammary gland is controlled by various hormonal and genetic signals [reviewed in 1], [ 2]

Using a genetic knock-out mouse model, we identified a key role for the epithelial-specific Ets transcription factor, Elf5, in the development of the mammary gland during pregnancy [3]. The complete lack of *Elf5* resulted in a lack of the extraembryonic ectoderm which led to early embryonic death [4]. Mice carrying one functional copy of the *Elf5* gene (*Elf5^+/−^*) were viable. However, unlike their wildtype littermates, *Elf5^+/−^* females were unable to support their offspring due to a pregnancy-associated mammary gland developmental defect, which prohibited these dams from lactating [3]. *Elf5* expression was downregulated in the *Prlr^+/−^* mammary gland while *Prlr* expression remained unchanged in the *Elf5^+/−^* mammary gland [3], indicating that Elf5 acts downstream of the Prlr in the Prl signalling cascade. This was later confirmed using a genetic complementation approach whereby ectopic expression of *Elf5* in the *Prlr*-null mammary epithelium was sufficient to rescue the developmental defect observed in the *Prlr*-null mice [5]. While it is known that Elf5 is able to activate the promoter of the milk protein gene *whey acidic protein* (*Wap*) [6], the mechanisms employed by Elf5 to induce its effect on mammary epithelial cell proliferation and differentiation are largely unknown. Here, we have used transcript profiling to investigate the transcriptional changes that occur as a result of *Elf5* haploinsufficiency as a first step in determining the mechanisms of Elf5 action and the targets of this transcription factor. We show that mammary gland development in the *Elf5^+/−^* female mouse is delayed during pregnancy due to the delayed increase in Elf5 protein expression in these glands. We identify a number of potential Elf5 target genes including *Muc4*, which we show is directly regulated by the binding of Elf5 to its promoter.

## Methods

### Mice

All work performed using mice as part of this study was approved by the appropriate Monash University Animal Ethics Committee and as such, all animal work followed the committee's guidelines and procedures. Mice were housed in a windowless room with controlled temperature (22°C±2°C), on a 12 hour light and dark cycle and were fed *ad libitum*. Pregnancy was determined by the presence of a vaginal plug and the day of detection designated 0.5 days post coitum (dpc). Mammary glands were collected between 11am and 1pm to control for circadian Prl release. Mice targeted at the *Elf5* locus were generated previously in our laboratory [3] and backcrossed for more than 10 generations onto a C57Bl/6 genetic background.

### Microarray analysis

Microarray analysis of the *Elf5^+/−^* mammary glands was performed using a common reference experimental design. Abdominal mammary glands (minus the associated lymph nodes) of 2 mice were pooled (to ensure enough material was obtained from virgin samples) and total RNA extracted using a RNeasy maxi kit (Qiagen) according to the manufacturer's instructions. RNA for use as the reference was extracted from eight embryonic (17.5dpc) C57Bl/6 mice using the same method. The RNA samples were sent to the Adelaide Microarray Facility for the remainder of the processing (see website for processing protocols: http://www.microarray.adelaide.edu.au). Five stages of mammary gland development (virgin, 8.5dpc, 10.5dpc, 14.5dpc and 16.5dpc), in two distinct genotypes (*Elf5^+/+^* and *Elf5^+/−^*) were examined in triplicate. A total of 30 array slides was used to examine 3 independent pooled RNA samples per genotype per timepoint. The slides chosen for this study were 22,000 element Compugen array chips which were spotted with single stranded oligonucleotides of 60 base pair length. Processed slides were scanned and the resulting images analysed using Digital Genome (Molecularware). Data analysis was performed using the Genespring software suite (Agilent). All data produced is MIAME compliant and has been deposited in the NCBI Gene Expression Omnibus database under series accession number GSE23373 (http://www.ncbi.nlm.nih.gov/geo/query/acc.cgi?acc=GSE23373).

### RNA extraction and gene expression analysis

Total RNA was extracted from mammary glands using Trizol reagent (Invitrogen) according to the manufacturer's instructions. RNA was treated with DNase (Promega) before being converted to cDNA using Superscript III (Invitrogen) and random primers (Promega). Quantitative real-time RT-PCR was performed on samples using TaqMan Gene Expression Assays (ABI) specific for Elf5 and Muc4. Relative expression was determined using the ΔΔC_T_ method [reviewed in 7], with the expression of the genes of interest first being normalised to the level of 18S expression.

### Protein extraction and Western blotting

Snap frozen tissue was homogenised in tissue lysis buffer (50 mM Hepes pH 7.5, 100 mM NaCl, 1 mM EDTA, 10% (v/v) glycerol, 0.5% (v/v) Nonidet-P40, Roche protease inhibitor cocktail tablet), and centrifuged to remove any insoluble material. Protein (50 µg) was separated using SDS-PAGE. Fractionated proteins were transferred to polyvinylidene fluoride (PVDF) membrane (Immobilon-P, Millipore) and membranes blocked for 1 hour at room temperature with 5% skim milk/TBST, before incubating with primary antibody overnight at 4°C. All secondary antibodies were conjugated to horseradish peroxidase and obtained from Dako. Membranes were incubated with SuperSignal West Pico chemiluminescent substrate (Pierce), after which they were exposed to Hyperfilm ECL film (Amersham Pharmacia), to detect chemiluminescence. Antibodies used in this study: anti-Elf5 (N-20) (Santa Cruz, catalogue number SC-9645); anti-mouse milk specific proteins (Accurate Chemical and Scientific Corporation, catalogue number YNRMMSP). Blotting for Muc4 was performed as described previously [8]

### Recombinant ELF5 protein preparation

Recombinant ELF5 protein was made by cloning a truncated *ELF5* cDNA into the *Bam*HI/*Sac*II sites of the pQE-30 (Qiagen) vector enabling the production of a truncated Elf5 (Δ33) protein fused to a (HIS)_6_ tag. Primers used to amplify the truncated cDNA were as follows: hELF5Δ33BamHI_F: 5′ CGTAGGATCCGCCTTTGAGCATCAGACAG 3′ and hELF5Δ33SacI_R: 5′ CATGGAGCTCAGCTTGTCTTCCTGCCACCC 3′. TALON beads (Clontech) were used to purify the protein as per the manufacturer's instructions.

### Cell transfection and reporter assays

The human breast carcinoma cell line T47D (ATCC) was grown in 1x RPMI with 10% (v/v) FCS plus 1% (v/v) penicillin/streptomycin and maintained in culture at 37°C, 5% CO_2._ T47D cells were plated in a 24 well plate at a density of 5×10^4^ cells/well in complete media. These cells were left to settle at 37°C, 5% CO_2_ for 24 hours before being transiently transfected with a total of 500 ng of DNA using the FuGENE6 transfection reagent according to the manufacturer's instructions (using a 1∶3 DNA:FuGENE ratio). Cells were transfected with a *MUC4* promoter-luciferase reporter construct (wild-type or mutated) [9], [10] along with an ELF5 expression vector and a β-galactosidase expression construct that was used as a measure of transfection efficiency. The cells were left for 48 hours before being harvested for luciferase and β-galactosidase assays. Luciferase reporter gene assays were performed using the Constant light signal kit (Roche) according to the manufacturer's instructions. β-galactosidase assays were performed by incubating a portion of the cell lysate with an equal volume of β-galactosidase sample buffer (0.1 M β-mercaptoethanol, 2 mM MgCl_2_, 88 µM Na_2_HPO_4_.2H_2_O, 90 µM NaH_2_PO_4_.2H_2_O, 4.5 µM 2-nitrophenyl-β-D-galactopyraniside), at 37°C for 1–2 hours or until colour developed. Luciferase and β-galactosidase assays were read on a Flourostar Optima plate reader (BMG). Absorbance for the β-galactosidase assay was read at 415 nm. Luciferase readings were normalised by division with the corresponding β-galactosidase reading.

### Isolation and extraction of nuclei

Snap frozen mammary glands, excised from 16.5dpc mice, were crushed in a mortar and pestle under liquid nitrogen, to a fine powder which was then mixed with ice-cold buffer A (10 mM Hepes pH 7.9, 1.5 mM MgCl_2_, 10 mM KCl, 500 µM PMSF, 500 µM DTT −900 µl per gland). The suspension was centrifuged, and the pellet resuspended in 20 µl of buffer A with the addition of Nonidet-P40 (to 0.15% v/v) and incubated on ice for 10 mins. The sample was centrifuged and the pellet resuspended in 15 µl of ice-cold buffer C (20 mM Hepes pH 7.9, 420 mM NaCl, 1.5 mM MgCl_2_, 0.2 mM EDTA, 25% (v/v) glycerol, 500 µM PMSF) before centrifugation and transfer of the supernatant to a fresh tube containing 40 µl of ice-cold buffer D (10 mM Hepes pH 7.9, 50 mM KCl, 0.2 mM EDTA, 20% (v/v) glycerol, 500 µM PMSF, 0.5 mM DTT).

### Electrophoretic mobility shift assay (EMSA)

Short double stranded oligonucleotides were end-labelled with (γ-^32^P)dATP (Perkin-Elmer) using T4 polynucleotide kinase (Promega), according to the manufacturer's instructions. When examining ELF5 binding to the *MUC4* promoter, an oligonucleotide containing the *Ets* site predicted at −216 was used as a probe 5′CCACCAGGAAAGAAAACACCG 3′. A mutant sequence where GGAA was changed to AAAA was also used. Nuclear extract (4 µg) or recombinant ELF5 protein (200 ng), with 1x bandshift binding buffer (40% (v/v) glycerol, 10 mM EDTA, 50 mM DTT, 100 mM Tris pH 7.5, 1 M NaCl, 1 mg/ml BSA) and a 100-fold molar excess of unlabelled competitor oligonucleotide were combined in a total volume of 20 µl. After 30 mins at room temperature, 30,000 counts per minute (cpm) of radiolabelled oligonucleotide probe was added and incubation continued for an additional 30 mins. Reactions were electrophoresed on polyacrylamide gels which were dried under vacuum onto blotting paper and then exposed to X-ray film.

## Results

### Transcript profiling indicated that pregnancy-associated development of the *Elf5^+/−^* mammary gland was delayed

**Figure 1:**
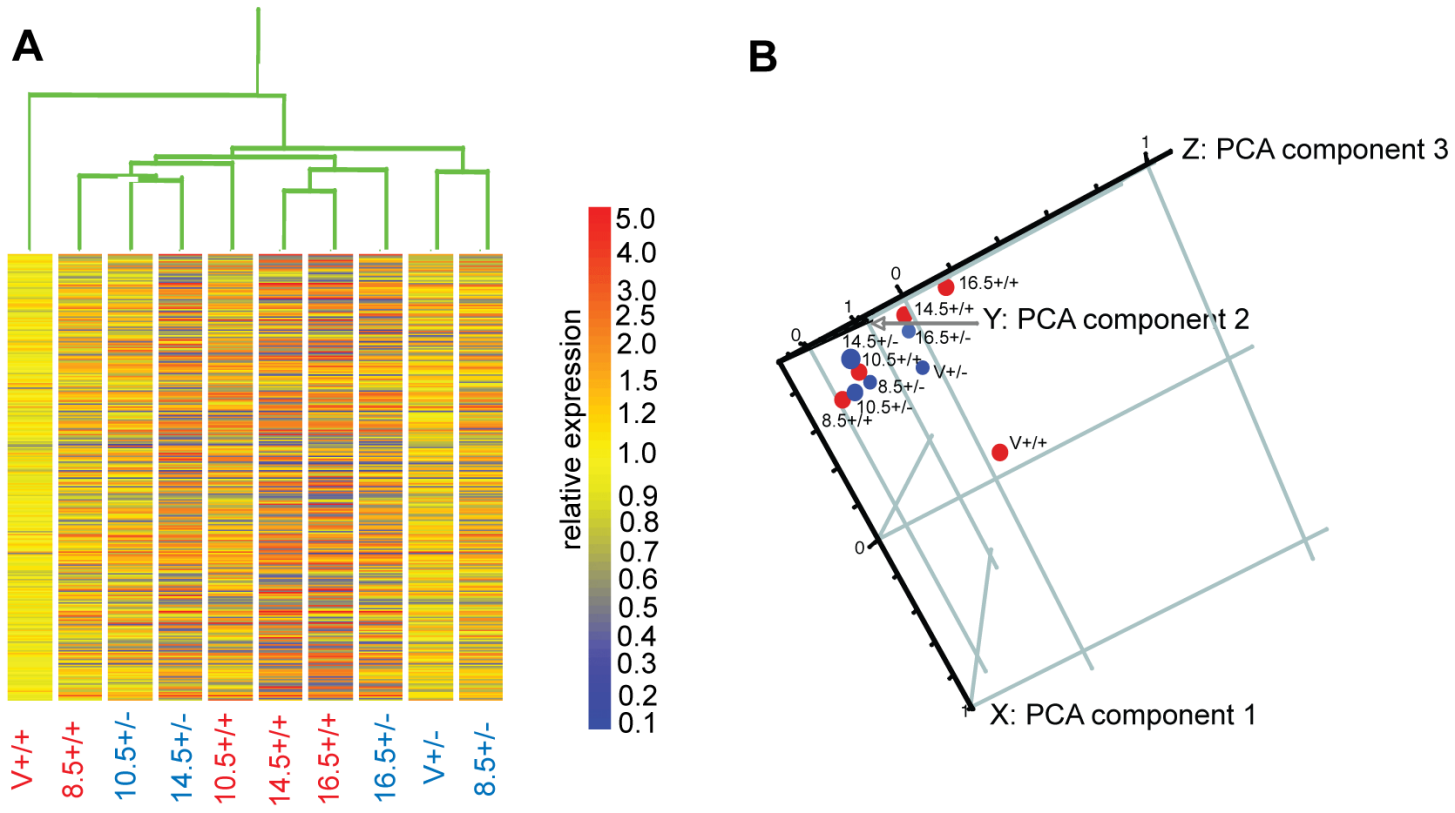
Comparing the Transcriptional profiles of *Elf5^+/+^* and *Elf5^+/−^* mammary glands. **A.** A condition dendrogram was generated within the Genespring software suite using an average linking clustering algorithm and a Spearman correlation similarity measure. Individual genes are represented by horizontal bars, which are coloured according to their expression level relative to the virgin Elf5^+/+^ (V+/+) sample. **B.** Principal components analysis was performed in Genespring on all 10 conditions examined by microarray. Conditions are plotted on a 3-dimensional scatter plot according to their variance from the first 3 components. The conditions were normalised to the V+/+ condition.

The changes in gene expression that occur as a result of the loss of one *Elf5* allele were investigated by transcript profiling. Having previously observed that the morphological development of the *Elf5^+/−^* mammary gland appears stunted [3], we sought to examine this potential developmental delay by transcriptional profiling. Profiles were generated for both the *Elf5^+/+^* and *Elf5^+/−^* mammary glands at 5 stages of mammary development (virgin, 8.5dpc, 10.5dpc, 14.5dpc and 16.5dpc). To examine the similarities between the global transcriptional profiles of each of the 10 experimental conditions (five stages of mammary gland development in 2 genotypes), a dendrogram (Figure 1a) was generated within Genespring using an average linking clustering algorithm and a Spearman correlation similarity measure.

The virgin wildtype mammary gland was placed on its own node of the dendrogram while all the other conditions were clustered on a separate arm. The *Elf5^+/−^* virgin and 8.5dpc samples were placed together on a separate node suggesting that they were most similar to one another, and more similar to the other pregnant conditions rather than to the *Elf5^+/+^* virgin condition. Notably, the *Elf5^+/−^* 10.5dpc and 14.5dpc conditions were clustered with the wildtype 8.5dpc sample, between the wildtype virgin and wildtype 10.5dpc samples, indicating that the expression profile of the 10.5dpc and 14.5dpc heterozygous gland was most similar to an earlier timepoint in the wildtype gland. Not surprisingly, the 14.5dpc and 16.5dpc wildtype conditions were most similar to one another. The *Elf5^+/−^* 16.5dpc condition was placed on a separate node, but adjacent to the wildtype 14.5dpc and 16.5dpc conditions, suggesting that the gene expression profile of the *Elf5^+/−^* 16.5dpc mammary gland had ‘caught up’ with the wildtype to some extent, although obvious gene expression differences remained.

A principal components analysis (PCA) was also performed on the dataset to examine the relationships between the transcriptional profiles of the experimental conditions (Figure 1b). A three-dimensional scatter plot mapping the experimental conditions by the first three principal components showed that the *Elf5*+/+ conditions are separated based upon whether they originated from a pregnant animal or not i.e. the virgin *Elf5*+/+ condition was placed alone, whereas the 4 conditions representing pregnant *Elf5*+/+ animals were clustered together. The 4 pregnant conditions showed increasingly greater variance from the first component in accordance with the stage of pregnancy. The separation of the wildtype pregnant conditions according to the stage of pregnancy also occurred in the direction of the third component. Here, the later the stage of pregnancy they represented the closer they were placed to 1 on the axis of the third component.

Supporting the condition dendrogram, the virgin *Elf5^+/−^* sample was positioned closer to the pregnant samples of both genotypes than to the wildtype virgin sample. The *Elf5^+/−^* 14.5dpc condition was placed closest to the 8.5dpc and 10.5dpc wildtype conditions suggesting that it had greatest similarity to the wildtype mammary gland at earlier stages of pregnancy. The 16.5dpc *Elf5^+/−^* condition was positioned closest to the 14.5dpc wildtype sample indicating that the expression profile of the *Elf5^+/−^* gland is becoming more similar to a more developed gland after 14.5dpc. It should be noted however that morphologically and functionally the *Elf5^+/−^* gland remains underdeveloped compared to the wildtype gland [3].

### Expression of milk proteins is delayed in the *Elf5^+/−^* mammary gland

**Figure 2:**
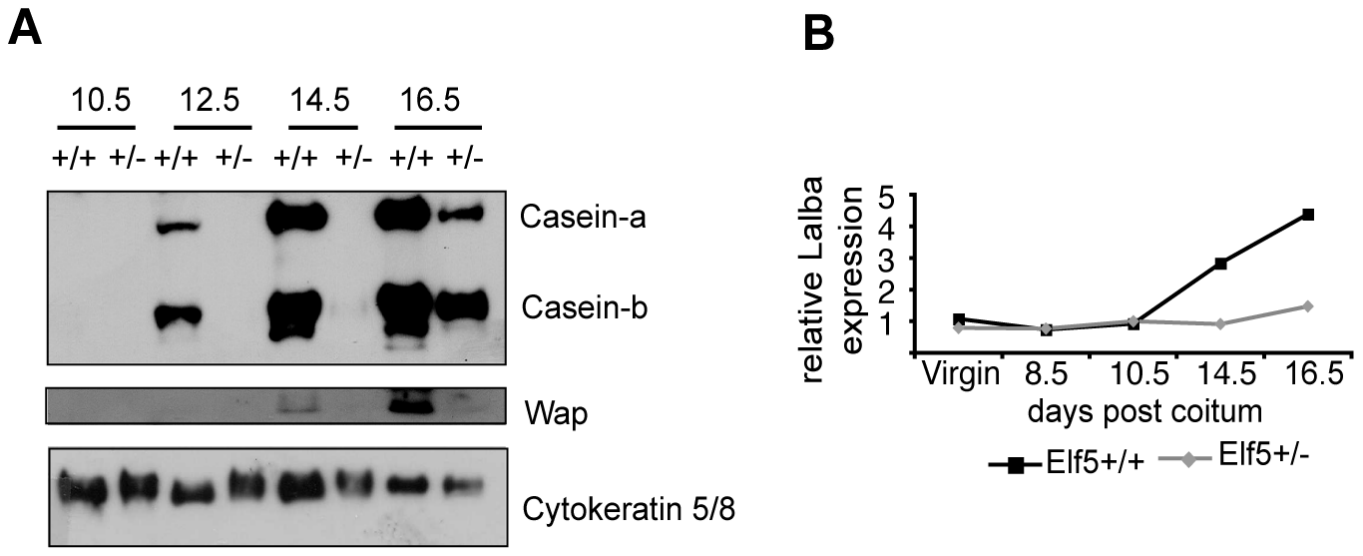
Differentiation of the *Elf5^+/−^* mammary gland during pregnancy is delayed. **A.** Western blot analysis of the milk proteins α-casein, β-casein and Wap in the mammary glands of *Elf5^+/+^* and *Elf5^+/−^* mice. **B.** Lactalbumin (Lalba) expression in the mammary glands of *Elf5^+/+^* and *Elf5^+/−^* mice measured by microarray analysis. Expression is shown relative to the virgin *Elf5^+/+^* sample.

Milk protein expression in the mammary gland can be used as a measure of differentiation. Expression of α-casein and β-casein proteins was first evident at 12.5dpc in the *Elf5*+/+ mammary gland, while Wap appeared at 14.5dpc (Figure 2a). In contrast, in the *Elf5^+/−^* mammary gland, expression of α- and β-caseins was not apparent until 16.5dpc. This result supports the notion that at 14.5dpc the *Elf5^+/−^* mammary gland is most similar to a very early stage wildtype gland, while at 16.5dpc the *Elf5^+/−^* gland is more like a gland that has entered the differentiation program. Indeed, the milk protein expression profile of the *Elf5^+/−^* 16.5dpc gland is similar to the wildtype 12.5dpc. gland.

Chronologically, the milk protein gene *α-lactalbumin* is the last milk protein gene to be expressed during pregnancy-associated mammary gland development [11]. The expression of *α-lactalbumin* has been used to determine whether a mammary gland has progressed from the first stage of lactogenesis known as the secretory initiation phase to the second stage, the secretory activation phase [12]. To determine whether the *Elf5^+/−^* mammary gland had proceeded through secretory initiation, we examined the level of *α-lactalbumin* expression in the *Elf5^+/−^* mammary gland in our microarray experiment (Figure 2b). Unlike the wildtype gland, at 14.5dpc there was no significant increase in *α-lactalbumin* expression in the *Elf5^+/−^* gland and furthermore, the static expression level of *α-lactalbumin* in the *Elf5^+/−^* gland suggested that the *Elf5^+/−^* gland had not proceeded through secretory initiation by 16.5dpc.

### Pregnancy associated increase of Elf5 expression is delayed in the *Elf5^+/−^* mammary gland

**Figure 3:**
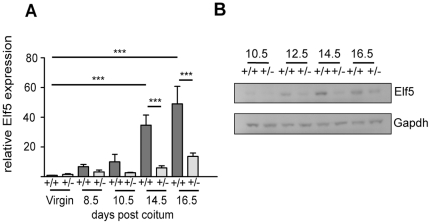
Elf5 expression profile during pregnancy associated mammary gland development. **A.** *Elf5* mRNA expression in in the mammary glands of *Elf5^+/+^* and *Elf5^+/−^* mice measured by qRT-PCR. Expression is shown relative to the average expression of *Elf5* in the *Elf5^+/+^* virgin samples. ***p<0.001. n = 3. **B.** Western blot analysis of Elf5 protein expression in the mammary glands of *Elf5^+/+^* and *Elf5^+/−^* mice during pregnancy.

To examine the correlation between *Elf5* expression levels and the gene expression profiles observed we measured *Elf5* mRNA and protein in *Elf5^+/+^* and *Elf5^+/−^* mammary glands at various stages of pregnancy (Figure 3). In the *Elf5* ^+/+^ mammary gland *Elf5* mRNA expression increased during the course of pregnancy, reaching a 49 fold induction in expression by 16.5dpc compared to its expression in the wildtype virgin gland (Figure 3a). Others have shown a similar Elf5 expression pattern during pregnancy-associated mammary gland development in C57Bl/6 x 129SVPas mice [5]. Expression of *Elf5* remained relatively low in the *Elf5^+/−^* mammary glands throughout early pregnancy but began to increase at the later timepoints (Figure 3a). In the *Elf5^+/−^* gland at 14.5dpc, *Elf5* expression increased by 5.94 fold with respect to the virgin wildtype gland, compared with 34.72 fold in the *Elf5^+/+^* gland (p<0.001). Likewise, at 16.5dpc *Elf5* expression in the *Elf5^+/−^* mammary gland had only increased by 13.69 fold (with respect to the virgin wildtype condition), whereas in the pregnant wildtype mammary gland at this stage, *Elf5* expression was increased by 49.08 fold (p<0.001). In the wildtype mammary gland, Elf5 protein was detected at all time points examined and its expression increased throughout pregnancy as previously described (Figure 3b) [3], [5]. Similar to its mRNA expression pattern, Elf5 protein was detected in all *Elf5^+/−^* mammary gland samples, however the levels of Elf5 expression remained comparable to that in the 10.5dpc *Elf5^+/−^* mammary gland until 16.5dpc where it increased slightly (Figure 3b).

### Identification of potential Elf5 target genes

To identify potential Elf5 targets, gene expression changes due to Elf5 heterozygosity were examined at each individual timepoint. Lists of the gene expression changes and the gene ontology analysis of these changes can be found in supplementary Tables S1, S2, S3, S4, S5, S6, S7, S8, S9, S10, S11, S12, S13, S14 with accompanying descriptions in supplementary files S1 and S2. We also used clustering algorithms to organise our data. This type of analysis assembles genes with common patterns of expression into groups, called clusters. Our rationale for choosing clustering was that genes grouped according to their similar expression patterns were likely to be co-regulated. By clustering our data set and identifying the cluster containing Wap, a known Elf5 target gene [6], we anticipated identifying other Elf5 targets.

**Figure 4:**
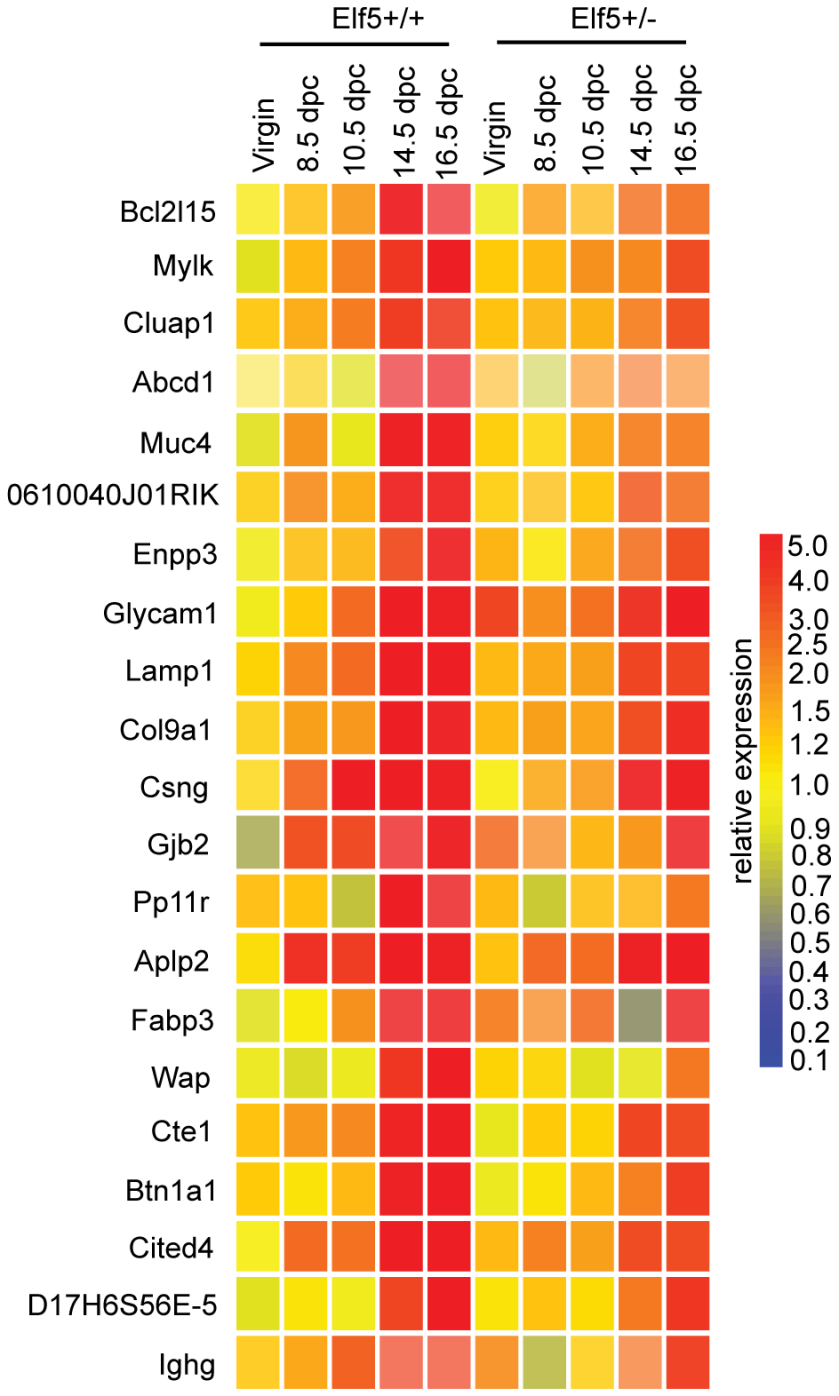
Genes that clustered with the known Elf5 target gene Wap. Expression profiles of the 23 genes that consistently clustered with *Wap* using 2 clustering algorithms, in the mammary glands of *Elf5^+/+^* and *Elf5^+/−^* mice. Gene expression is shown relative to expression in the virgin *Elf5^+/+^* condition.

Employing two commonly used algorithms, K-means and self organising maps (SOMs), we searched for the genes that clustered with *Wap*. Independent K-means clustering was performed 10 times, the first time with a K value of 5, after which the K value was increased by 5 each time until 50 clusters was reached. Of the 10 K-means analyses performed, K = 15 produced the highest explained variability (70.435%) and for this reason was used in the remainder of our analysis. We also generated multiple SOMs (4×4, 4×5, 4×6, 5×5, 5×6 and 6×6), with the 6×6 SOM generating the highest explained variability (79.419%). We identified the clusters from each of the 15 cluster K-means and the 6×6 SOM that contained *Wap* and compared the two lists to identify 23 common genes (Figure 4). The expression of all 23 of these genes was downregulated at all timepoints in the pregnant *Elf5^+/−^* gland compared to the pregnant wildtype gland.

Next we used the DAVID functional annotation clustering tool (http://david.abcc.ncifcrf.gov/home.jsp) [13] to determine whether any gene ontology classification was over-represented in the 23 genes that consistently clustered with *Wap* (Table 1). The gene ontology (GO) terms signal, extracellular space, cellular lipid metabolism, lipid metabolism and glycoprotein were all identified as being significantly enriched in the list with each having a p-value less than 0.05. The GO terms signal and extracellular space defined under annotation cluster 1, represented 34.78% of the 23 genes and included *Csng*, *Lamp1*, *Col9a1*, *Wap*, *Btn1a1*, *Glycam1*, *Aplp and Pp11r*. The terms cellular lipid metabolism and lipid metabolism defined under annotation cluster 2, represented 17.39% of the genes and included *Cte1*, *Lrat*, *Fabp3*, *Alox12e*. The term glycoprotein was also significantly enriched (annotation cluster 3) and represented the genes *Lamp1*, *Col9a1*, *Btn1a1*, *Glycam1*, *Aplp* and *Ighg*.

**Table 1:** Functional annotation clustering of the 23 genes that clustered with Wap.

GO Term	Number of genes	% of the 23 genes represented	p-value
*Annotation cluster 1*			
Signal	7	30.43%	0.0023
Extracellular space	8	34.78%	0.0068
*Annotation cluster 2*			
Cellular lipid metabolism	4	17.39%	0.0073
Lipid metabolism	4	17.39%	0.012
*Annotation cluster 3*			
Glycoprotein	6	26.09%	0.0234

### Identification of *Ets* binding sites in the upstream regions of potential Elf5 target genes

We hypothesised that the promoter of a gene whose expression is directly regulated by Elf5 would contain an *Ets* binding site – and more specifically, an *Ets* site with a specific flanking sequence reported to be preferred by Elf5 [5′(A/C)GGAA(G/A)(T/G)(A/G)NNC 3′] [14]. The predicted promoter regions (1000 bp upstream of the transcriptional start site) of 20 of the 23 genes clustered with *Wap* were obtained using EZretrieve. (http://siriusb/umdnj.edu:18080/EZRetrieve/index.jsp) and Promoser (http://biowulf.bu.edu/zlab/PromoSer). The upstream sequences for the genes *Acbd7*, *Ighg* and *Gm566* were not identified by these programs. Putative promoter sequences were searched using the Transcriptional Element Search System (TESS), which is accessible via the web-based Baylor College of Medicine (BCM) Search Launcher (http://searchlauncher.bcm.tmc.edu/). Of the 20 promoter sequences searched, 5 (*Wap*, *Muc4*, *Col9a1*, *Pp11r* and *Lamp1*) contained at least one putative *Ets* binding site. We chose to investigate *Muc4* as a potential direct transcriptional target of Elf5 since *Muc4* has a role in the pregnant mammary gland in rodents and in humans and dysregulated expression of *MUC4* has been associated with breast cancer [15]. Moreover, MUC4 is transcriptionally regulated by another Ets factor, PEA3 [9]. In addition, the predicted *Ets* sites at nucleotide positions −216 and −1613 (where numbering is relative to the initiating ATG) of the human *MUC4* promoter are almost identical to the preferred *Elf5* binding site, with only the 3′ nucleotide differing from the predicted *Elf5* preferred sequence.

### *Muc4* expression is decreased in the *Elf5^+/−^* mammary gland

**Figure 5:**
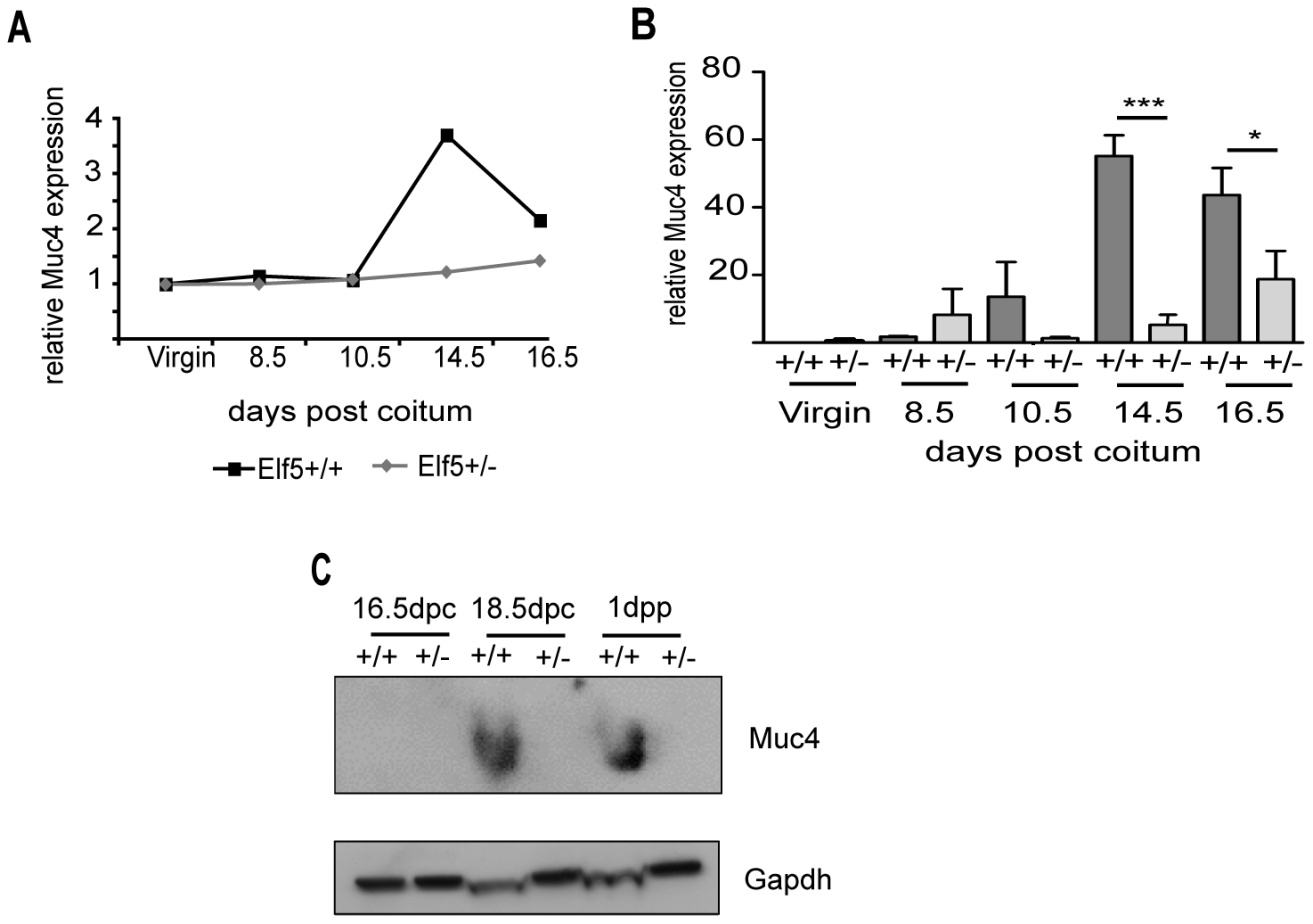
Examining *Muc4* as a potential Elf5 target gene. **A.** *Muc4* mRNA expression in the mammary glands of *Elf5^+/+^* and *Elf5^+/−^* mice measured by microarray analysis. Expression is shown relative to the virgin *Elf5^+/+^* condition. **B.** *Muc4* expression in the mammary glands of *Elf5^+/+^* and *Elf5^+/−^* mice measured by qRT-PCR. Expression is shown relative to the average expression of *Muc4* in the virgin *Elf5^+/+^* samples. ***p<0.001; *p<0.05. n = 3. **C**. Western blot examining Muc4 protein expression in *Elf5^+/+^* and *Elf5^+/−^* mammary glands.

The *Muc4* expression pattern observed in the microarray analysis (Figure 5a) was confirmed by quantitative real time RT-PCR on RNA samples distinct from those used in the microarray experiments (Figure 5b). While there was no significant difference in *Muc4* expression between the two genotypes in the virgin, 8.5dpc or 10.5dpc glands, a 10 fold reduction in *Muc4* expression was observed at 14.5dpc in the *Elf5^+/−^* gland compared to the wildtype (55.16 fold in the wildtype compared with 5.26 fold in *Elf5^+/−^*; p<0.001). A significant 2.3 fold decrease in *Muc4* expression was observed in the *Elf5^+/−^* gland at 16.5dpc compared with the 16.5dpc wildtype gland (43.65 fold in the wildtype gland compared with 18.76 fold in *Elf5^+/−^*; p<0.05). Correspondingly, while Muc4 protein increased dramatically at 18dpc and was sustained at 1dpp in the *Elf5^+/+^* mammary gland, Muc4 protein was undetectable in the *Elf5^+/−^* mammary glands at these times (Figure 5c).

### ELF5 can activate the *MUC4* promoter via the *Ets* binding site at −216

**Figure 6:**
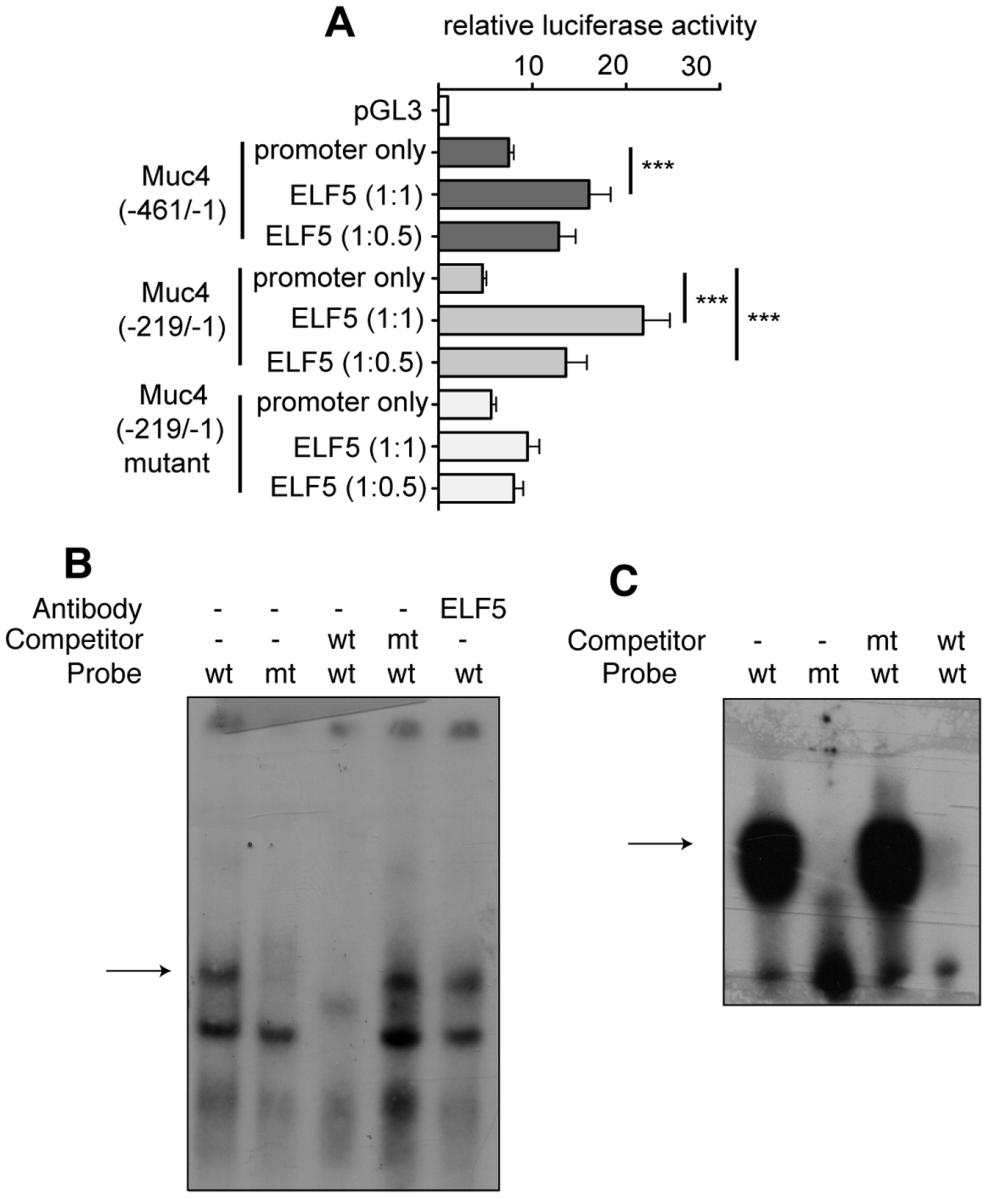
ELF5 directly regulates MUC4 promoter activity. **A**. The effect of ELF5 expression on *MUC4* promoter activity was tested using *MUC4* promoter-*luciferase* reporter gene assays with different regions of the *MUC4* promoter. Cells were transfected with either the promoter construct alone or with the promoter construct plus an ELF5 expression vector at molar ratios of 1∶1 or 1∶0.5 (promoter-*luciferase* vector:ELF5 expression vector). Luciferase expression is shown relative to the expression of the pGL3 promoterless vector. *** p<0.001; **p<0.01. n = 3. **B.** EMSA was used to determine whether Elf5 could bind the −216 *Ets* site within the *MUC4* promoter. Binding was observed (arrow) in lane 1 when a nuclear extract from a 16.5dpc *Elf5^+/+^* mammary gland was incubated with a radioactively labelled oligonucleotide encompassing the −216 *Ets* site from the *MUC4* promoter (wt). The binding observed was not evident when the *Ets* site in the probe was mutated (mt) (lane 2), indicating that the upper band represents specific binding to the *Ets* sequence. Binding to the wt probe was competed off with a 100-fold molar excess of unlabelled wt oligo (lane 3) but not with mt oligo (lane 4). Binding to the wt probe was not shifted with the addition of anti-ELF5 antibody (lane 5). **C.** The EMSA from (A) was performed again using a Δ33 ELF5 recombinant protein in place of the nuclear extract. The truncated recombinant ELF5 protein bound the labelled wt oligo (lane 1), but not the mt oligo (lane 2). Binding to the wt oligo could not be competed off with the mt oligo (lane 3), but was successfully competed off using an excess of unlabelled wt oligo (lane 4).

The full-length proximal h*MUC4* promoter (−461/−1) and a truncated proximal promoter (−219/−1) were used to drive a *luciferase* reporter gene [10]. The proximal promoter contains a predicted *ELF5*-preferred *ETS* binding site at position −216, in addition to a predicted *ETS* binding site at −349 [14]. The human breast carcinoma cell line T47D was used since it expresses *MUC4* [16] and therefore contains all the factors required for *MUC4* expression. Cells were transfected with the promoter constructs alone, or co-transfected with an ELF5 expression plasmid [6]. The full-length proximal *MUC4* promoter triggered a 7.49 fold increase in luciferase activity over the empty pGL3 vector (Figure 6a). Co-expression of ELF5 in these cells at a 1∶1 molar ratio (ELF5:MUC4 promoter) resulted in a significant increase in promoter activity (16.06 fold over the promoterless vector; p<0.001). Luciferase expression driven by the truncated *MUC4* proximal promoter increased 4.69 fold above the baseline of the promoterless vector and significant increases in luciferase activity were observed when ELF5 was over-expressed in these cells at a 0.5∶1 or 1∶1 (ELF5:promoter) molar ratio (p<0.001) indicating that ELF5 was acting on the *MUC4* promoter.

To confirm that ELF5 was activating the *MUC4* promoter via the preferred *ELF5* binding site at nucleotide position −216, the promoter-*luciferase* reporter experiments were repeated with a *MUC4* −219/−1 promoter-*luciferase* construct in which the *Ets* binding site at −216 had been mutated (GGAA was changed to AAAA) [9]. This −216/−1 mutant construct showed promoter activity equivalent to that of the wildtype −216/−1 region, indicating that basal *MUC4* expression in T47D cells was not dependant on the *Ets* binding site at position −216. However, over-expression of ELF5 failed to induce activity of the mutated *MUC4* promoter (Figure 6a), indicating that ELF5 was acting on the *Ets* binding site at nucleotide position −216.

### ELF5 binds the *Ets* binding site at −216 in the *MUC4* promoter

To confirm that ELF5 regulated *MUC4* expression by directly binding its target sequence, we performed electrophoretic mobility shift assays (EMSA). Two bands were evident upon incubation of the wildtype probe with the d16.5 pregnant mammary gland nuclear extract (Figure 6b, lane 1) indicating binding to the probe. When a probe containing the mutated *Ets* binding site was used only the lower band was evident (lane 2) suggesting that the higher band represented specific binding. Binding to the wildtype probe could be competed off by the addition of a 100-fold molar excess of a non-radiolabelled wildtype probe to the nuclear extract (lane 3) while the addition of an excess of mutated non-radiolabelled probe had no effect on binding activity (lane 4) confirming that binding to the −216 *Ets* site was specific. To determine whether it was Elf5 in the nuclear extract binding to the site we performed a supershift assay by adding an anti-Elf5 antibody to the nuclear extract/probe mix. The addition of this antibody had no effect on the mobility of the probe/protein complex (lane 5). However, it is possible that this anti-Elf5 antibody does not function in a supershift assay.

Since we could not determine the identity of the protein in the nuclear extract binding the *MUC4* promoter, we performed an EMSA using recombinant ELF5 protein. Full length recombinant ELF5 protein does not bind DNA efficiently due to the presence of a negative regulatory domain at the amino end of the protein [17], therefore a truncated ELF5 protein lacking 33 amino acids from the amino terminus was produced. The Δ33 ELF5 protein was able to bind the wildtype probe (Figure 6c, lane 1) but was unable to bind the mutated probe (lane 2), indicating that the binding to the wildtype probe was specific. Incubation with a 100-fold molar excess of the unlabelled mutated probe did not alter binding to the wildtype probe (lane 3) but addition of unlabelled wildtype probe competed off all binding (lane 4).

## Discussion

Elf5 plays a major role in pregnancy-associated mammary gland development. Re-expression of Elf5 in the epithelium of the Prlr^−/−^ mammary gland restored mammary gland development during pregnancy [5], revealing that *Elf5* acts downstream of the Prlr, presumably as a Stat5 target gene [1], [2]. However for the most part, the mechanism underpinning Elf5 function remains unclear. We used transcript profiling to examine the transcriptional changes induced by the loss of one *Elf5* allele, as a first step to defining this mechanism.

### Transcript profiling revealed a delay in the development of the *Elf5* ^+/−^ mammary gland during pregnancy

It has been proposed that Elf5 plays a major role in the co-ordination of proliferation, differentiation and apoptosis in the mammary epithelial cell compartment [18]. Our results, and the phenotype of the Elf5-over-expressing mouse [19], support the notion that Elf5 is essential for mammary epithelial cell differentiation. We have shown that the global gene expression profile of the mammary gland shifts upon robust expression of Elf5 protein and coincides with the first expression of milk proteins. We observed delayed synthesis of milk proteins in the *Elf5^+/−^* gland where increased expression of Elf5 protein was delayed until 16.5dpc, suggesting that these mammary glands are not entering the differentiation phase of the developmental program until this time.

With the exception of *Wap* [6], the direct transcriptional targets of Elf5 in the mammary gland are unknown. Clustering analysis allowed us to generate a list of 23 genes with expression profiles similar to *Wap*, with six already known to play roles in the mammary gland (*Csng* [*20*]; *Glycam1* [21]; *Muc4* [22]; *Fabp3* [23]; *Btn1a1* [24] and *Cx26* [25]). Functional annotation of the 23 gene cluster revealed a significant enrichment for genes associated with signalling and the extracellular space, lipid metabolism and glycoproteins.

Of the 23 genes identified through clustering analysis, only 5 contained at least one *Ets* binding site in the promoter region analysed. A number of possible explanations exist. Firstly, the remaining genes may not be direct targets of Elf5 but may be regulated by factors which are themselves dependent on Elf5. Secondly, it is possible that Elf5 forms a complex with one or more other proteins and that this complex binds the promoter regions of target genes via the binding sites of the non-Ets proteins within the complex. Thirdly, the possibility exists that *Ets* binding sites involved in the regulation of these genes are located within a region outside of the 1000 base pairs searched in our study.

### *MUC4* gene expression is directly regulated by ELF5

Muc4 is a glycoprotein located within the membrane of secretory epithelial cells [26] and its expression is dysregulated in breast cancer [15]. Here we have shown that *Muc4* expression is significantly down-regulated in the *Elf5^+/−^* mammary gland compared to the wildtype gland at 14.5dpc and 16.5dpc. In the T47D breast cancer cell line, exogenous addition of ELF5 was able to induce expression from the *MUC4* promoter. Furthermore, we have shown that ELF5 is able to directly bind the *MUC4* promoter via a preferred ELF5 binding site.

Interestingly, the binding site used by ELF5 to regulate *MUC4* expression is the same site used by another Ets factor, PEA3, to regulate *MUC4* expression in the pancreas [9], in HC11 mouse mammary epithelial cells and in MAT-B1 and MAT-C1 rat mammary tumour cells [27], suggesting some functional redundancy. However, *Elf5 and Pea3* are expressed at different stages of post-natal mammary gland development and the expression pattern of *Muc4* in the pregnant mammary gland most closely resembles that of Elf5. The expression of both *Elf5* and *Muc4* is induced at mid pregnancy, peaking early in lactation, and diminishing during involution [reviewed in 3], [5], [26], whereas Pea3 protein expression is highest during puberty and during early pregnancy [reviewed in 28]. It therefore seems most likely that *Muc4* expression in the pregnant mammary gland is regulated by Elf5 and not Pea3.

In rat mammary epithelial cells, Muc4 acts as a ligand for the ErbB2 receptor [29]. It has been suggested that a Muc4/ErbB2 complex is involved in the maintenance and survival of alveolar epithelial cells during pregnancy and that the downregulation of *Muc4* and *ErbB2* expression during involution plays a role in the initiation of apoptosis [30]. Although Muc4 protein is absent in *Elf5^+/−^* mammary glands during pregnancy, we have previously shown that there is no increase in apoptosis in *Elf5^+/−^* mammary glands [3].

The formation of a complex between Muc4 and its receptor ErbB2 on the surface of mammary epithelial cells triggers ErbB2 autophosphorylation on the tyrosine residue at position 1248 [31]. In polarised epithelial cells, ErbB2 resides on the basolateral surface of the cell along with other ErbB receptors. The association of Muc4 with ErbB2 leads to the translocation of ErbB2 from the basolateral to the apical surface of the cell [31]. This repositioning effectively separates ErbB2 from its usual heterodimerisation partner - ErbB3. ErbB2/ErbB3 dimers are strong inducers of cellular proliferation [32], therefore the separation of these proteins prevents downstream proliferation signals from these receptors. The separation of ErbB2 from ErbB3 induced by Muc4 is thought to trigger the ‘switch’ from the proliferative phase to the differentiation phase in mammary epithelial cells [29], [31].

The absence of Muc4 protein in the *Elf5^+/−^* gland would abrogate the formation of the Muc4/ErbB2 complex and the subsequent phosophorlation of ErbB2. Therefore it is likely that the switch from proliferation to differentiation is not induced in *Elf5^+/−^* mammary glands. However we did not observe excessive proliferation in the *Elf5* ^+/−^ mammary glands. Rather, these glands contained fewer alveolar structures than the wildtype gland. Therefore there must be other mechanisms involved in the cessation of mammary epithelial cell proliferation. Our data is consistent with Elf5 acting as a controller of a switch to differentiation, since robust expression of Elf5 protein in the *Elf5^+/−^* mammary gland resulted in a marked shift in the transcriptional profile of the gland and the first expression of milk proteins – definitive markers of differentiation. We propose that Elf5 controls this switch partly via its regulation of *Muc4*.

It is not unprecedented for an Ets family member to be involved in the control of the cellular cues that drive proliferation versus differentiation. Pea3 is a positive regulator of Muc4 expression in pancreatic cancer cells, whilst also acting as a negative regulator of ErbB2 [9]. Via its control of the balance between relative expression levels of Muc4 and ErbB2, Pea3 is thought to promote pancreatic cancer cell differentiation [9]. There is the possibility that the delicate balance of proliferation and differentiation may be a mechanism often driven by Ets factors.

Our hypothesis that Elf5 regulates the switch from mammary epithelial cell proliferation to differentiation is supported by the report of a mouse in which ectopic over-expression of Elf5 in the mammary gland led to a reduction in epithelial proliferation and forced differentiation of the mammary epithelium [19]. This resulted in the expression of numerous milk proteins in non-pregnant mammary glands [19]. This phenotype may have occurred via a direct upregulation of *Muc4* by Elf5 and the subsequent termination of proliferative signals generated by ErbB2 and ErbB3 dimers on the basolateral surface of the epithelial cells.

The novel findings of our study give insight into a newly defined role for Elf5 in the pregnant mammary gland. We hypothesise that via the direct regulation of *Muc4* expression, Elf5 co-ordinates the switch between mammary epithelial cell proliferation and differentiation during pregnancy, making it a master regulator of cellular processes in this organ.

## Supporting Information

File S1 Genes with significantly different expression in the *Elf5^+/−^* mammary gland compared with the *Elf5^+/+^* mammary gland. Tables S1-S10.
(0.02 MB DOC)

File S2 Functional Annotation Clustering - Tables S11-S14.
(0.03 MB DOC)

Table S1 Genes upregulated in *Elf5^+/−^* virgin mammary gland compared to *Elf5^+/+^* virgin mammary gland.
(0.03 MB DOC)

Table S2 Genes downregulated in *Elf5^+/−^* virgin mammary gland compared to *Elf5^+/+^* virgin mammary gland.
(0.03 MB DOC)

Table S3 Genes upregulated in *Elf5^+/−^* mammary gland compared to *Elf5^+/+^* mammary gland at 8.5dpc.
(0.03 MB DOC)

Table S4 Genes downregulated in *Elf5^+/−^* mammary gland compared to *Elf5^+/+^* mammary gland at 8.5dpc.
(0.03 MB DOC)

Table S5 Genes upregulated in *Elf5* ^+/−^ mammary gland compared to *Elf5* ^+/+^ mammary gland at 10.5dpc.
(0.03 MB DOC)

Table S6 Genes downregulated in *Elf5^+/−^* mammary gland compared to *Elf5^+/+^* mammary gland at 10.5dpc.
(0.03 MB DOC)

Table S7 Genes upregulated in *Elf5^+/−^* mammary gland compared to *Elf5^+/+^* mammary gland at 14.5dpc.
(0.03 MB DOC)

Table S8 Genes downregulated in *Elf5^+/−^* mammary gland compared to *Elf5^+/+^* mammary gland at 14.5dpc.
(0.04 MB DOC)

Table S9 Genes upregulated in *Elf5^+/−^* mammary gland compared to *Elf5^+/+^* mammary gland at 16.5dpc
(0.04 MB DOC)

Table S10 Genes downregulated in *Elf5^+/−^* mammary gland compared to *Elf5^+/+^* mammary gland at 16.5dpc.
(0.07 MB DOC)

Table S11 Functional annotation clustering of genes dysregulated in the *Elf5^+/−^* virgin mammary gland.
(0.03 MB DOC)

Table S12 Functional annotation clustering of genes downregulated in the *Elf5^+/−^* mammary gland at 14.5dpc.
(0.04 MB DOC)

Table S13 Functional annotation clustering of genes downregulated in the *Elf5^+/−^* mammary gland at 16.5dpc.
(0.03 MB DOC)

Table S14 Functional annotation clustering of genes upregulated in the *Elf5^+/−^* mammary gland at 16.5dpc.
(0.03 MB DOC)
