# Supplementary material for: Transcript Profiling of Elf5+/− Mammary Glands during Pregnancy Identifies Novel Targets of Elf5
Source: PLoS One. 2010 Oct 7;5(10):e13150. doi: 10.1371/journal.pone.0013150 (PMC2951341; doi:10.1371/journal.pone.0013150)
Supplement: File S1 — Genes with significantly different expression in the Elf5+/− mammary gland compared with the Elf5+/+ mammary gland. Tables S1-S10. (0.02 MB DOC) [file pone.0013150.s001.doc]

**Genes with significantly different expression in the *Elf5*^+/-^ mammary gland compared with the *Elf5*^+/+^ mammary gland. Tables S1-S10.**

Lists of genes whose expression was up or down-regulated in the *Elf5*^+/-^ mammary gland were generated using Genespring. An arbitrary cut-off of 2 fold level of expression difference was chosen. Gene lists were subjected to a parametric t-test where variances were not assumed equal (Welch t-test). No multiple correction testing was performed.
