# Supplementary material for: Transcript Profiling of Elf5+/− Mammary Glands during Pregnancy Identifies Novel Targets of Elf5
Source: PLoS One. 2010 Oct 7;5(10):e13150. doi: 10.1371/journal.pone.0013150 (PMC2951341; doi:10.1371/journal.pone.0013150)
Supplement: File S2 — Functional Annotation Clustering - Tables S11-S14. (0.03 MB DOC) [file pone.0013150.s002.doc]

**Functional Annotation Clustering - Tables S11-S14.**

Functional annotation clustering was performed on the lists of genes whose expression was found to be statistically dysregulated in the *Elf5*^+/-^ mammary gland compared to the *Elf5*^+/+^ gland. This was performed using the DAVID ontological tool which examines gene lists for enrichment of biological themes or ontology groups. The gene list of interest was compared against a background gene list of all genes represented on the Compugen array slide used.

Due to the small number of significant gene changes, no enrichment of ontological terms was found for the genes dysregulated in the *Elf5*^+/-^ mammary gland at 8.5dpc and 10.5dpc. Similarly, no enrichment of ontological terms was uncovered in the genes whose expression was upregulated in the *Elf5*^+/-^ mammary gland at 14.5dpc.

Please note, due to the small number of genes found to be dysregulated in the virgin mammary gland, all genes (those whose expression increased along with those whose expression decreased) were used to look for enrichment of biological themes.
