# Supplementary material for: Transcript Profiling of Elf5+/− Mammary Glands during Pregnancy Identifies Novel Targets of Elf5
Source: PLoS One. 2010 Oct 7;5(10):e13150. doi: 10.1371/journal.pone.0013150 (PMC2951341; doi:10.1371/journal.pone.0013150)
Supplement: Table S1 — Genes upregulated in Elf5+/− virgin mammary gland compared to Elf5+/+ virgin mammary gland. (0.03 MB DOC) [file pone.0013150.s003.doc]

**Table S1**. **Genes upregulated in *Elf5*^+/-^ virgin mammary gland compared to *Elf5*^+/+^ virgin mammary gland**

| Accession number | Gene Name | Description | P value |
| --- | --- | --- | --- |
| NM_019640 | Pitpnb | Phosphatidylinositol transfer protein, beta | 0.0464 |
| U20264 |  | Mus musculus clone 1.5B/C/D LB9 mRNA, 3'UTR, partial sequence. | 0.0192 |
| AK011820 | Fbxo31 | F-box only protein 5 | 0.0162 |
| U62675 | H2b-616 | Mus musculus histone H2b-616 (H2b-616), complete cds. | 0.0125 |
| NM_026331 | Slc25a37 | Mitochondrial solute carrier protein | 0.0114 |
| AK011437 |  | RIKEN cDNA 4930420N18 gene | 0.00367 |
