# Supplementary material for: Transcript Profiling of Elf5+/− Mammary Glands during Pregnancy Identifies Novel Targets of Elf5
Source: PLoS One. 2010 Oct 7;5(10):e13150. doi: 10.1371/journal.pone.0013150 (PMC2951341; doi:10.1371/journal.pone.0013150)
Supplement: Table S2 — Genes downregulated in Elf5+/− virgin mammary gland compared to Elf5+/+ virgin mammary gland. (0.03 MB DOC) [file pone.0013150.s004.doc]

**Table S2**. **Genes downregulated in *Elf5*^+/-^ virgin mammary gland compared to *Elf5*^+/+^ virgin mammary gland**

| **Accession number** | **Gene Name** | **Description** | **P value** |
| --- | --- | --- | --- |
| AK005423 | Abp1 | Amiloride binding protein 1 (amine oxidase, copper-containing) | 0.0438 |
| NM_008161 | Gpx3 | Glutathione peroxidase 3 | 0.0385 |
| NM_008522 | Ltf | Lactotransferrin | 0.0303 |
| AK005050 | Dhdh | Dihydrodiol dehydrogenase (dimeric) | 0.018 |
| NM_011491 | Stc2 | Stanniocalcin 2 | 0.00847 |
| NM_009701 | Aqp5 | Aquaporin 5 | 0.00734 |
| NM_009194 | Nkcc1/Slc12a2 | Solute carrier family 12, member 2 | 0.0049 |
