# Supplementary material for: Transcript Profiling of Elf5+/− Mammary Glands during Pregnancy Identifies Novel Targets of Elf5
Source: PLoS One. 2010 Oct 7;5(10):e13150. doi: 10.1371/journal.pone.0013150 (PMC2951341; doi:10.1371/journal.pone.0013150)
Supplement: Table S3 — Genes upregulated in Elf5+/− mammary gland compared to Elf5+/+ mammary gland at 8.5dpc. (0.03 MB DOC) [file pone.0013150.s005.doc]

**Table S3. Genes upregulated in *Elf5*^+/-^ mammary gland compared to *Elf5*^+/+^ mammary gland at 8.5dpc**

| **Accession number** | **Gene Name** | **Description** | **P value** |
| --- | --- | --- | --- |
| NM_007504 | Atp2a1 | ATPase, Ca++ transporting, cardiac muscle, fast twitch 1 | 0.0413 |
| NM_011620 | Tnnt3 | Troponin T3, skeletal, fast | 0.0166 |
