# Supplementary material for: Transcript Profiling of Elf5+/− Mammary Glands during Pregnancy Identifies Novel Targets of Elf5
Source: PLoS One. 2010 Oct 7;5(10):e13150. doi: 10.1371/journal.pone.0013150 (PMC2951341; doi:10.1371/journal.pone.0013150)
Supplement: Table S4 — Genes downregulated in Elf5+/− mammary gland compared to Elf5+/+ mammary gland at 8.5dpc. (0.03 MB DOC) [file pone.0013150.s006.doc]

**Table S4**. **Genes downregulated in *Elf5*^+/-^ mammary gland compared to *Elf5*^+/+^ mammary gland at 8.5dpc**

| **Accession number** | **Gene Name** | **Description** | **P value** |
| --- | --- | --- | --- |
| AB072039 | Fae | ELOVL family member 6, elongation of long chain fatty acids (yeast) | 0.0453 |
| NM_011814 | Fxr2 | Fragile X mental retardation gene 2, autosomal homolog | 0.0166 |
