# Supplementary material for: Transcript Profiling of Elf5+/− Mammary Glands during Pregnancy Identifies Novel Targets of Elf5
Source: PLoS One. 2010 Oct 7;5(10):e13150. doi: 10.1371/journal.pone.0013150 (PMC2951341; doi:10.1371/journal.pone.0013150)
Supplement: Table S5 — Genes upregulated in Elf5 +/− mammary gland compared to Elf5 +/+ mammary gland at 10.5dpc. (0.03 MB DOC) [file pone.0013150.s007.doc]

**Table S5.** **Genes upregulated in *Elf5*+/- mammary gland compared to *Elf5*+/+ mammary gland at 10.5dpc**

| **Accession number** | **Gene Name** | **Description** | **P value** |
| --- | --- | --- | --- |
| NM_008476 | Krt6a | Keratin complex 2, basic, gene 6a | 0.017 |
| AF067834 | Casp8 | Caspase 8 | 0.0143 |
