# Supplementary material for: Transcript Profiling of Elf5+/− Mammary Glands during Pregnancy Identifies Novel Targets of Elf5
Source: PLoS One. 2010 Oct 7;5(10):e13150. doi: 10.1371/journal.pone.0013150 (PMC2951341; doi:10.1371/journal.pone.0013150)
Supplement: Table S6 — Genes downregulated in Elf5+/− mammary gland compared to Elf5+/+ mammary gland at 10.5dpc. (0.03 MB DOC) [file pone.0013150.s008.doc]

**Table S6. Genes downregulated in *Elf5*^+/-^ mammary gland compared to *Elf5*^+/+^** mammary gland at 10.5dpc

| **Accession number** | **Gene Name** | **Description** | **P value** |
| --- | --- | --- | --- |
| M29018 |  | Mouse ribosomal protein L19-17 (rpL19-17) processed pseudogene. | 0.0456 |
| AK019457 |  | RIKEN cDNA 3732412D22 gene | 0.043 |
| NM_007785 | Csng | Casein gamma | 0.0398 |
| NM_008318 | Ibsp | Integrin binding sialoprotein | 0.0308 |
| NM_008578 | Mef2b | Myocyte enhancer factor 2b | 0.0159 |
| AK003570 | Actn2 | Actinin alpha 2 | 0.0155 |
| NM_011814 | Fxr2 | Fragile X mental retardation gene 2, autosomal homolog | 0.00609 |
