# Supplementary material for: Transcript Profiling of Elf5+/− Mammary Glands during Pregnancy Identifies Novel Targets of Elf5
Source: PLoS One. 2010 Oct 7;5(10):e13150. doi: 10.1371/journal.pone.0013150 (PMC2951341; doi:10.1371/journal.pone.0013150)
Supplement: Table S7 — Genes upregulated in Elf5+/− mammary gland compared to Elf5+/+ mammary gland at 14.5dpc. (0.03 MB DOC) [file pone.0013150.s009.doc]

**Table S7**. **Genes upregulated in *Elf5^+/-^* mammary gland compared to *Elf5^+/+^*mammary gland at 14.5dpc**

| **Accession number** | **Gene Name** | **Description** | **P value** |
| --- | --- | --- | --- |
| NM_026178 | Mmd | Monocyte to macrophage differentiation-associated | 0.0472 |
| AF206329 | Polydom | Polydomain protein | 0.0362 |
| U29489 |  | Mus musculus retinoic acid receptor alpha-regulated E3 protein gene, promoter region | 0.0305 |
| AK010765 | Bag4 | BCL2-associated athanogene 4 | 0.0297 |
| NM_011994 | Abcd2 | ATP-binding cassette, sub-family D (ALD), member 2 | 0.0291 |
| NM_008161 | Gpx3 | Glutathione peroxidase 3 | 0.0169 |
