# Supplementary material for: Transcript Profiling of Elf5+/− Mammary Glands during Pregnancy Identifies Novel Targets of Elf5
Source: PLoS One. 2010 Oct 7;5(10):e13150. doi: 10.1371/journal.pone.0013150 (PMC2951341; doi:10.1371/journal.pone.0013150)
Supplement: Table S8 — Genes downregulated in Elf5+/− mammary gland compared to Elf5+/+ mammary gland at 14.5dpc. (0.04 MB DOC) [file pone.0013150.s010.doc]

**Table S8**. **Genes downregulated in *Elf5*^+/-^ mammary gland compared to *Elf5*^+/+^ mammary gland at 14.5dpc**

| **Accession number** | **Gene Name** | **Description** | **P value** |
| --- | --- | --- | --- |
| NM_008128 | Gjb6 | Gap junction membrane channel protein beta 6 | 0.048 |
| NM_009158 | Mapk10 | Mitogen activated protein kinase 10 | 0.0476 |
| NM_011402 | Slc34a2 | Solute carrier family 34 (sodium phosphate), member 2 | 0.0448 |
| NM_013602 | Mt1 | Metallothionein 1 | 0.0405 |
| L19564 | Slc6a2 | Solute carrier family 6, member 2 | 0.0403 |
| AF014453 | Gm566 | Gene model 566, (NCBI) | 0.0353 |
| NM_009263 | Spp1 | Secreted phosphoprotein 1 | 0.0341 |
| AF357363 |  | Mus musculus clone MBII-98 C/D box snoRNA | 0.032 |
| L13622 | Mat1a | Methionine adenosyltransferase I, alpha | 0.0266 |
| AF255061 | Lrat | Lecithin-retinol acyltransferase (phosphatidylcholine-retinol-O-acyltransferase) | 0.0263 |
| NM_016972 | Slc7a8 | Solute carrier family 7 (cationic amino acid transporter, y+ system), member 8 | 0.0256 |
| X90779 | psH2B | M.musculus psH2B gene. | 0.0252 |
| M97632 | Slc6a12 | Solute carrier family 6 (neurotransmitter transporter, betaine/GABA), member 12 | 0.0246 |
| AF338222 | Chrdl2 | Chordin-like 2 | 0.0221 |
| NM_008125 | Gjb2 | Gap junction membrane channel protein beta 2 | 0.0216 |
| BC014728 | Tmem38a | Transmembrane protein 38a | 0.021 |
| NM_008902 | Pp11r | Placental protein 11 related | 0.0178 |
| NM_010174 | Fabp3 | Fatty acid binding protein 3, muscle and heart | 0.01 |
| AK006270 | 1700023E05Rik | RIKEN cDNA 1700023E05 gene | 0.00239 |
