# Supplementary material for: Transcript Profiling of Elf5+/− Mammary Glands during Pregnancy Identifies Novel Targets of Elf5
Source: PLoS One. 2010 Oct 7;5(10):e13150. doi: 10.1371/journal.pone.0013150 (PMC2951341; doi:10.1371/journal.pone.0013150)
Supplement: Table S9 — Genes upregulated in Elf5+/− mammary gland compared to Elf5+/+ mammary gland at 16.5dpc (0.04 MB DOC) [file pone.0013150.s011.doc]

**Table S9**. **Genes upregulated in *Elf5*^+/-^ mammary gland compared to *Elf5*^+/+^ mammary gland at 16.5dpc**

| **Accession number** | **Gene Name** | **Description** | **P value** |
| --- | --- | --- | --- |
| NM_018800 | Syt6 | Synaptotagmin 6 | 0.0494 |
| AF203898 | Neb | Mus musculus nebulin mRNA, partial cds. | 0.0485 |
| NM_010052 | Dlk1 | Delta-like 1 homolog (Drosophila) | 0.0479 |
| NM_009659 | Alox12b | Arachidonate 12-lipoxygenase, 12R type | 0.0423 |
| U20264 | Copg2as2; Lb9; Mit1 | Mus musculus clone 1.5B/C/D LB9 mRNA, 3'UTR, partial sequence. | 0.0423 |
| NM_021477 | A2bp1 | Ataxin 2 binding protein 1 | 0.0376 |
| NM_009258 | Spink3 | Serine protease inhibitor, Kazal type 3 | 0.0368 |
| NM_007710 | Ckm | Creatine kinase, muscle | 0.0258 |
| AK020939 | 6030443J06Rik | RIKEN cDNA 1700030G06 gene | 0.0162 |
| NM_010168 | F2 | Coagulation factor II | 0.0156 |
| AK004221 | 1110050K14Rik | RIKEN cDNA 1110050K14 gene | 0.00993 |
| NM_025285 | Stmn2 | Stathmin-like 2 | 0.00974 |
| AB021969 | Cpn1 | Carboxypeptidase N, polypeptide 1 | 0.00938 |
| NM_008877 |  | Mus musculus plasminogen (Plg), mRNA | 0.00769 |
| NM_011359 | Sftpc | Surfactant associated protein C | 0.00739 |
| NM_007482 | Arg1 | Arginase 1, liver | 0.00523 |
| NM_013456 | Actn3 | Actinin alpha 3 | 0.00285 |
