# Supplementary material for: Transcript Profiling of Elf5+/− Mammary Glands during Pregnancy Identifies Novel Targets of Elf5
Source: PLoS One. 2010 Oct 7;5(10):e13150. doi: 10.1371/journal.pone.0013150 (PMC2951341; doi:10.1371/journal.pone.0013150)
Supplement: Table S10 — Genes downregulated in Elf5+/− mammary gland compared to Elf5+/+ mammary gland at 16.5dpc. (0.07 MB DOC) [file pone.0013150.s012.doc]

**Table S10**. **Genes downregulated in *Elf5*^+/-^ mammary gland compared to *Elf5*^+/+^ mammary gland at 16.5dpc**

| **Accession number** | **Gene Name** | **Description** | **P value** |
| --- | --- | --- | --- |
| AF218416 | Ttpa | Tocopherol (alpha) transfer protein | 0.0471 |
| AF015205 |  | Immunoglobulin heavy chain variable region precursor | 0.0468 |
| Mm.16716 |  | Mus musculus lysosomal membrane glycoprotein 1 (Lamp1), mRNA | 0.042 |
| AK014708 | Cdw92/Slc44a1 | RIKEN cDNA 9130223C08 gene | 0.0389 |
| U55664 | Igk-V1 | Immunoglobulin kappa chain variable 5 (V5 family) | 0.0386 |
| AF014453 | Gm566 | Gene model 566, (NCBI) | 0.0376 |
| AF073933 |  | Mus musculus WW domain binding protein 14 mRNA, partial sequence. | 0.0368 |
| NM_007785 | Csng | Casein gamma | 0.0321 |
| X75536 | Igk-V8 | Immunoglobulin kappa chain variable 8 (V8) | 0.032 |
| AJ251891 | BH151 | Gene model 1502, (NCBI) | 0.0314 |
| BC011467 | BC011467 | CDNA sequence BC011467 | 0.0311 |
| NM_008128 | Gjb6/Cx30 | Gap junction membrane channel protein beta 6/Connexin 30 | 0.0309 |
| AK013949 | 3110001I20Rik | Hypothetical protein C630011I23 | 0.0305 |
| NM_011709 | Wap | Whey acidic protein | 0.0298 |
| NM_011082 | Pigr | Polymeric immunoglobulin receptor | 0.0279 |
| AY007202 | Muc4 | Mucin 4 | 0.0277 |
| U21587 |  | Similar to Ig kappa chain V region (C8.5) - mouse (fragment) | 0.0261 |
| AK008963 | Ogfrl1 | Opioid growth factor receptor-like 1 | 0.0253 |
| D29939 | Igk-V1 | Immunoglobulin kappa chain variable 21 (V21) | 0.0252 |
| NM_009864 | Cdh1 | Cadherin 1 | 0.0251 |
| U07814 | Igk-V8 | Immunoglobulin kappa chain variable 8 (V8) | 0.0236 |
| NM_011977 | Slc27a1 | Solute carrier family 27 (fatty acid transporter), member 1 | 0.0213 |
| AB072976 | Fads1 | Fatty acid desaturase 1 | 0.0189 |
| M25565 |  | Mouse Ig active H-chain mRNA, V-region, subgroup IIB, clone L06. | 0.0189 |
| NM_008340 | Igfals | Insulin-like growth factor binding protein, acid labile subunit | 0.0181 |
| M27587 | Igk-V1 | Immunoglobulin kappa chain variable 5 (V5 family) | 0.0173 |
| NM_010679 | Lalba | Lactalbumin, alpha | 0.0152 |
| NM_030565 | BC004044 | CDNA sequence BC004044 | 0.0114 |
| AB006831 |  | Mus sp. mRNA for anti-pseudouridine monoclonal antibody light chain variable region, partial cds. | 0.0108 |
| BC006914 | Lzf | Leucine zipper domain protein | 0.0106 |
| NM_008529 | Ly6e | Lymphocyte antigen 6 complex, locus E | 0.00969 |
| NM_019477 | Acsl4 | Acyl-CoA synthetase long-chain family member 4 | 0.00899 |
| U55462 | Ankrd12 | Ankyrin repeat domain 12 | 0.00683 |
| AK005560 | 1600029I14Rik | RIKEN cDNA 1600029I14 gene | 0.0065 |
| NM_013912 | Apln | Apelin | 0.00565 |
| NM_026345 | Mansc1 | MANSC domain containing 1 | 0.00463 |
| V00856 | Wap | Whey acidic protein | 0.0039 |
