# Supplementary material for: Transcript Profiling of Elf5+/− Mammary Glands during Pregnancy Identifies Novel Targets of Elf5
Source: PLoS One. 2010 Oct 7;5(10):e13150. doi: 10.1371/journal.pone.0013150 (PMC2951341; doi:10.1371/journal.pone.0013150)
Supplement: Table S11 — Functional annotation clustering of genes dysregulated in the Elf5+/− virgin mammary gland. (0.03 MB DOC) [file pone.0013150.s013.doc]

**Table S11: Functional annotation clustering of genes dysregulated in the *Elf5*^+/-^** virgin mammary gland

| **GO term** | **Number of genes represented** | **% of the 13 genes dysregulated in the virgin gland** | **P value** |
| --- | --- | --- | --- |
| *Annotation cluster 1* | | | |
| Transport | 7 | 53.84% | 0.0008 |
| Glycoprotein | 5 | 38.46% | 0.014 |
| Establishment of localization | 7 | 53.84% | 0.019 |
