# Supplementary material for: Transcript Profiling of Elf5+/− Mammary Glands during Pregnancy Identifies Novel Targets of Elf5
Source: PLoS One. 2010 Oct 7;5(10):e13150. doi: 10.1371/journal.pone.0013150 (PMC2951341; doi:10.1371/journal.pone.0013150)
Supplement: Table S12 — Functional annotation clustering of genes downregulated in the Elf5+/− mammary gland at 14.5dpc. (0.04 MB DOC) [file pone.0013150.s014.doc]

**Table S12: Functional annotation clustering of genes downregulated in the *Elf5*^+/-^** mammary gland at 14.5dpc

| **GO term** | **Number of genes represented** | **% of the 19 genes downregulated in the Elf5^+/-^ gland at 14.5dpc** | **P value** |
| --- | --- | --- | --- |
| *Annotation cluster 1* | | | |
| Porter activity | 4 | 21.05% | 9.81E-04 |
| Electrochemical potential driven transporter activity | 4 | 21.05% | 100E-03 |
| Amine transporter activity | 3 | 15.79% | 0.00023 |
| Symporter activity | 3 | 15.79% | 0.00045 |
| Carrier activity | 4 | 21.05% | 0.0110 |
| Plasma membrane | 6 | 31.58% | 0.0138 |
| Transporter activity | 6 | 31.58% | 0.0152 |
| Transmembrane region | 7 | 36.84% | 0.0305 |
| *Annotation Cluster 2* | | | |
| Cell communication | 4 | 21.05% | 2.97E-04 |
| Ossification | 3 | 15.79% | 0.00536 |
| Biomineral formation | 3 | 15.79% | 0.00536 |
| Bone remodeling | 3 | 15.79% | 0.00633 |
| Tissue remodeling | 3 | 15.79% | 0.00677 |
| Skeletal development | 3 | 15.79% | 0.01627 |
| Growth factor activity | 3 | 15.79% | 0.01785 |
| Focal adhesion | 3 | 15.79% | 0.03072 |
| Tissue development | 3 | 15.79% | 0.03300 |
| Organismal physiological process | 6 | 31.58% | 0.03550 |
