# Supplementary material for: Transcript Profiling of Elf5+/− Mammary Glands during Pregnancy Identifies Novel Targets of Elf5
Source: PLoS One. 2010 Oct 7;5(10):e13150. doi: 10.1371/journal.pone.0013150 (PMC2951341; doi:10.1371/journal.pone.0013150)
Supplement: Table S13 — Functional annotation clustering of genes downregulated in the Elf5+/− mammary gland at 16.5dpc. (0.03 MB DOC) [file pone.0013150.s015.doc]

**Table S13: Functional annotation clustering of genes downregulated in the *Elf5*^+/-^** mammary gland at 16.5dpc

| **GO term** | **Number of genes represented** | **% of the 37 genes downregulated in the Elf5^+/-^ gland at 16.5dpc** | **P value** |
| --- | --- | --- | --- |
| *Annotation cluster 1* | | | |
| Extracellular space | 10 | 35.71% | 0.00250 |
| Extracellular region | 10 | 35.71% | 0.00429 |
| Signal peptide | 9 | 32.14% | 0.00557 |
| Signal | 9 | 32.14% | 0.00821 |
| *Annotation cluster 2* | | | |
| Fatty acid metabolism | 3 | 10.71% | 0.01109 |
| *Annotation cluster 3* | | | |
| Transporter activity | 6 | 21.43% | 0.01524 |
