# Supplementary material for: Transcript Profiling of Elf5+/− Mammary Glands during Pregnancy Identifies Novel Targets of Elf5
Source: PLoS One. 2010 Oct 7;5(10):e13150. doi: 10.1371/journal.pone.0013150 (PMC2951341; doi:10.1371/journal.pone.0013150)
Supplement: Table S14 — Functional annotation clustering of genes upregulated in the Elf5+/− mammary gland at 16.5dpc. (0.03 MB DOC) [file pone.0013150.s016.doc]

**Table S14: Functional annotation clustering of genes upregulated in the Elf5^+/-^** mammary gland at 16.5dpc

| **GO term** | **Number of genes represented** | **% of the 17 genes upregulated in the Elf5^+/-^ gland at 16.5dpc** | **P value** |
| --- | --- | --- | --- |
| *Annotation cluster 1* | | | |
| Protease | 4 | 21.05% | 0.00927 |
| Extracellular region | 6 | 31.58% | 0.04354 |
| *Annotation cluster 2* | | | |
| Calcium ion binding | 6 | 31.58% | 8.08E-04 |
| Cation binding | 9 | 47.37% | 0.00286 |
| Metal ion binding | 9 | 47.37% | 0.00494 |
| Ion binding | 9 | 47.37% | 0.004941 |
